# Overweight or Obesity, Gender, and Age Influence on High School Students of the City of Toluca's Physical Fitness

**DOI:** 10.1155/2017/9546738

**Published:** 2017-08-06

**Authors:** Flor de Maria Cruz Estrada, Patricia Tlatempa Sotelo, Roxana Valdes-Ramos, José Aldo Hernández Murúa, Rafael Manjarrez-Montes-de-Oca

**Affiliations:** ^1^Facultad de Medicina, Universidad Autónoma del Estado de México, Toluca, MEX, Mexico; ^2^Facultad de Ciencias de la Conducta, Universidad Autónoma del Estado de México, Toluca, MEX, Mexico; ^3^Escuela Superior de Educación Física, Universidad Autónoma de Sinaloa, Culiacán, SIN, Mexico

## Abstract

**Material and Method:**

This is a prospective, cross-sectional, and correlational study with a probabilistic sampling in which 150 teenagers from three different high schools from the city of Toluca, Mexico, aged 15–17, were assessed.

**Objective:**

To determine if weight, age, and gender have an influence on physical fitness evaluated with the EUROFIT and ALPHA-FITNESS batteries.

**Results:**

Women have a higher overweight and obesity rate than men (3 : 1). Adolescents who have normal weight have regular physical fitness (74.9%). When comparing genders we found that men have a higher mean than women in the tests, except for skinfold thickness and waist circumference. Age was only correlated with the plate tapping test (*p* = 0.001). There are significant differences in the standing broad jump test and the Course-Navette of the EUROFIT and ALPHA-FITNESS batteries (*p* = 0.000).

**Conclusions:**

It is likely that regular physical activity, and not normal weight, helps generate healthy physical fitness. Male subjects had a higher mean than women, reporting a better physical fitness and more frequent physical activity.

## 1. Introduction

 Physical fitness is defined as the capability of an individual to carry out their everyday activities without excessive fatigue and with enough spare energy to enjoy their free time and to solve unusual [1] situations. It is also regarded as an integrative measure of most of the functions and structures that take part when doing exercise or any physical activity. Such functions are musculoskeletal, cardiorespiratory, haematocirculatory, endocrine-metabolic, and psychoneurological [1, 2].

Low physical fitness (PF) and physical inactivity (PI) have been on the increase at schools in the last five decades. Overweight/obesity affects 10% of students globally. However, this figure reaches 30% [3, 4] in Latin America [2, 3]. In Latin-American countries, only 15% of the 5–17-year-olds fulfil the everyday physical activity requirements (PA) [3], which represents a public health issue as far as nontransmissible chronic diseases in young population are concerned. It is argued that physical inactivity that starts at school tends to continue until adulthood [3] and that low physical fitness in kids and teenagers is a risk factor for future development of cardiovascular diseases at later stages in life [4].

There are plenty of studies which have measured the level of physical fitness in teenagers, among which are the following: European Fitness Test Battery (EUROFIT) [5], Actividad Física y Salud del Instituto Nacional de Educación Física de Cataluña (AFISAL-INEFC) [6, 7], and one of the latest Assessing Levels of Physical Activity and Fitness at population level (ALPHA-FITNESS) [8]. Such research outlines the importance of the components and parameters of physical fitness and its relation to health.

Personal data taken from the Feeding and Valuation of Teenagers Nutritional Status (AVENA) describe that the Spanish teenager population has excessively low physical fitness in comparison to teenagers from other countries. There is a shortage of similar studies in Mexico, especially those whose subjects are kids or teenagers, which raises the need for such studies in order to understand the Mexican situation, for this country is suffering the consequences of an epidemiological transition to chronic degenerative diseases. According to the Organization for Economic Cooperation and Development (OCDE 2010) Mexico is the first place in the world in overweight (30%) and the second in obesity (24%).

According to the Mexican Observatory of Noncommunicable Diseases (OMENT 2015, for its initials in Spanish), 32% of the population in the State of Mexico are overweight, and 20% have obesity problems, which predisposes this population to diseases such as diabetes mellitus type I (8% of the population older than 10 years), diabetes mellitus type II (73.4 deaths for every 100 thousand inhabitants), hypertension (17% of the population over 20 years old), and acute myocardial infarction and cerebrovascular events (13.9 deaths every 100 thousand inhabitants).

Despite the fact that chronic diseases and cardiovascular accidents happen after the fifth decade of life, scientific evidence indicates that the origins of a cardiovascular disease can be found in childhood and adolescence [8]. Thus, clinic monitoring is of the utmost importance in order to diagnose overweight and obesity in time and so that we are able to avoid problems such as health costs, inability and physical disability, and premature death (years potentially lost); so promoting a healthy and productive population that modifies future habits and passes on this knowledge to future generation is urgent.

The objective of this study was to determine if overweight, obesity, and fat percentage, as well as age and gender, have an influence on physical fitness, is evaluated with the components of the modified and extended EUROFIT and ALPHA-FITNESS battery tests.

## 2. Methods and Material

### 2.1. Design and Sampling

This is a cross-sectional, correlational, and prospective study with a randomized probabilistic sampling; it was carried out in Toluca City, State of Mexico, in one public and two private high schools, with a total sample of 204 Mexican teenagers, 54 of which were eliminated according to the established criteria, leaving a total of 150 (87 women) teenagers of ages varying from 15 to 17 years, who undertook the modified and extended EUROFIT and ALPHA-FITNESS battery tests.

The extended ALPHA-FITNESS is considered to be modified because the Tanner studies are omitted in women and men and so the equation to estimate fat mass (%) according to the pubertal stage is not considered either.

### 2.2. Intervention

The intervention was carried out for all semesters, both genders and both shifts, morning and evening of the schools. The parents of the students were dutifully informed about the purpose and protocol of the research and everybody gave their permission. The criteria for exclusion included the following aspects: chronic muscular skeletal diseases, physical inability or disability, medical prescription, pregnancy, and unsigned permissions. Elimination was due to having missed two evaluation days, disease, or injuries during the intervention. The project was carried out inside every school during school time in both shifts.

The research was carried out regarding the ethical rules stated in the declaration of Helsinki (AMA 2013) and the Mexican ruling for research in humans (first chapter, only chapter about the Ruling of the General Health Law for Health Research). This study has been peer reviewed and has been approved by the Research and Ethics Committee (document 15CE01720131119) of the Medical Sciences Research Centre of the Autonomous University of the State of Mexico (UAEMex).

### 2.3. Evaluation of Physical Fitness

#### 2.3.1. Anthropometric Assessment

It was carried out by standardised personnel, under the monitoring of two Level II specialists of the International Society for the Advancement of Kinanthropometry (ISAK). All the participants were evaluated in one room for men and one for women where the following data was obtained: weight (kg), stature (cm), body mass index (BMI: kg/m^2^), waist and hip circumferences (cm), waist-to-hip ratio (ICC: waist circumference/hip circumference), and skinfold thickness (bicipital, tricipital, and supraspinatus).

All participants were barefoot, standing, and with a minimum of clothes when the measures were done. For the weight measure the subjects were standing on the model 514 Tanita® electric weighing machine. The stature was measured with a model 206 SECA® stadiometer, with their heels, buttocks, and back against the wall in relation to the Frankfort plane. Finally, waist and hip circumferences were measured only once using the model CESCORF® anthropometric tape. Body mass index was obtained of the dividend of weight with kilograms divided by the stature in square meters. For the skinfolds, marks with a dermosensitive pen and a CESCORF® anthropometric tape were used. A Harpenden® skinfold caliper was used for the measures, by duplicate, and three times if there was an important variation between the first and the second test.

### 2.4. Physical Fitness Assessment

The components and protocols for every extended and modified EUROFIT [1, 9] and ALPHA-FITNESS [10–12] tests were considered. The subjects wore sports clothes (sports pants or short and *t*-shirt) and suitable footwear (tennis shoes). The tests were carried out inside the school sport facilities, in assigned spaces for the room tests, and in the physical activity area for the field tests.

A stereotypical box was used for the torso flexion test. Sitting on a flat surface, both legs spread, touching the box with their feet and keeping their body straight, the subject moved one hand above the other over the board in order to reach the farthest possible distance. Two tries were made. Only the maximum distance (cm) achieved was registered.

In the long jump test (LJ) a metric tape was placed on a flat horizontal surface. The subjects had to jump with both feet together, without running, and with their arms in movement to gain impulse. The distance was measured between the foot that was behind and the start line. Two tries were made. Only the maximum distance (cm) achieved was registered.

For hand strength, we used a digital Takei TKK5001 dynamometer (0–100 kgf range). The subjects applied the maximum hand strength in two alternative attempts with each hand. With a standardised position, the final result was the total of the measures of both attempts.

The 30-second abs test was carried out on a yoga rug on a flat surface. The subject was in the supine position with their knees flexed, arms next to their body, and palms touching the surface. One of the assessors shouted “Go!” and started the countdown. The other assessor held the ankles of the subject and counted the number of repetitions. There was a try and an attempt, registering the most quantity of repetitions made in 30 seconds.

Bent arm hang test was carried out with a pull-up bar for arm bending, standard position. There was only one attempt and the recorded time was the longest one (sec).

The flamingo balance test was carried out on a flat surface where a horizontal bar was placed. The subject stood on one foot (the same foot throughout all the test). One of the assessors was taking the time and noting the attempts the subject made, the test was carried out only once, and it finished in 60 seconds or at the subject's 10th attempt in less than 60 seconds. The number of attempts was registered.

The 10 × 5 meter shuttle run test took place on a flat surface in which two determinate lines were mapped out at 5 m. When the assessor signalled, the subject ran from one line to the other, ten times, that is, five times back and forth in the shortest amount of time possible. Two attempts were made, and the shortest time (sec.) was recorded.

For the 4 × 10 m shuttle run test the subjects ran back and forth between two ten-meter lines carrying a disc cone in the shortest amount of time they could. Two attempts were made and the shortest time (sec.) was recorded.

The tapping test was carried out over a table in which a central rectangle and two lateral, nonslip discs were placed. The subject placed a hand on the central rectangle, while he used the other hand to touch alternately each of the circles as fast as they could. One of the assessors noted the time and motivated the subject while the other assessor counted how many times the subject hit the circles. 25 unilateral hits were completed. Two attempts using each hand were made. The shortest time (sec.) was considered.

The aerobic capacity test was measured with an indirect, incremental, and maximum test. This was the 20-meter shuttle run test or Course-Navette test. Cones were used to mark the 20-meter track for the test. The POLAR® RC3 GPS HR gear, alongside with the thoracic tape, was used. The subject ran nonstop from one line to the other, turning when signalled by the recorded beeps. The initial speed was 8.5 km/hr and increased at 0.5 km/hr/min. The subject must step after the line before the next beep. The test finishes when the participant does not step two consecutive times after the line or when they are exhausted. The aerobic performance was carried out once. It was expressed according to the last stage that was reached.

## 3. Variables

In the* EUROFIT* battery all the components were taken into account and were carried out according to the protocol. The figures obtained in each variable for each participant were captured into the EUROFIT software. A categorical rating was obtained according to the age and gender of the participant for each test and finally another categorical rate for the general physical fitness. The categories are bad, average, good, remarkable, and excellent.

For the modified, extended* ALPHA-FITNESS* battery all aspects were considered except Tanner. According to the points obtained based on the values of manual reference of instructions, the results were grouped in five categories for age and gender groups: very low, low, average, high, and very high.

## 4. Statistical Analysis

The SPSS v. 23.0 for Windows (SPSS Inc., Chicago, USA.) was used for the codification and data analysis. Descriptive analysis was carried out (means and standard deviation). The normality of the data distribution was established for each of the variables through the Anderson-Darling. The significance level was set at *p* ≤ 0.05 and confidence interval 95%.

Spearman's rank correlation coefficient was used in both batteries for the age and gender variables versus the physical fitness corresponding variables of each battery. This test was not met in all the variables, so the nonparametric Kruskal Wallis test was a substitute for the ANOVA test.

For overweight, obesity, and fat percentage variables of the EUROFIT battery only the Spearman correlation was carried out. For the extended, modified ALPHA-FITNESS battery, the ANOVA test and the post hoc (Dunnett's) were also carried out. The homologue variable results of the EUROFIT and the extended modified ALPHA-TEST batteries were then compared through the equality of means test for related samples (Student's *t*-test).

## 5. Results

Of all the sample, 150 teenagers (87 women) who took part in the EUROFIT and the extended, modified ALPHA-FITNESS battery tests, ranging from 15 to 17 years old (mean ± standard deviation 16 ± 1.91 years), there were 111 subjects (74.7%) in the normal weight category; 12 of them (8%) were in the low weight category; 18 teenagers (12%) were overweight, and only 8 subjects (5.3%) suffered from obesity. Moreover, women presented a higher mean than men (Table 1). It is important to point out that all the subjects reported regular physical activity when in fact the result showed that 71.4% of men and 31.3% of women did regular physical activity.

Table 1 shows the descriptive data regarding gender and age with respect to the BMI and it is clear that most of the participants in the normal weight category were 15 years old (76.4%). The group of 16-year-olds is the one where most of the women (14.2%) suffer overweight and in group of 17-year-olds 9% suffer obesity. There are no overweight and obesity men in the group of those who are 17 years old.

## 6. EUROFIT Battery

The estimate for Spearman's rank correlation coefficient (Table 2) was carried out for the gender and age variables versus EUROFIT physical fitness variables and it can be seen that the gender correlates with the flamingo balance test, the torso flexion, standing broad jump, stature, weight, and overall assessment. Age correlates with plate tapping test.

The single factor ANOVA test was carried out; the homogeneity of variance was not met for the flamingo balance test, the torso flexion test, and the standing broad test; thus the Kruskal Wallis parametric test was used for these variables (Table 3). With this test we may conclude that there are significant differences: gender does have an influence in the response of the flamingo balance test (*p* = 0.006), torso flexion (*p* = 0.025), and standing broad test (*p* = 0.000), as well as for the height test (*p* = 0.000), weight (*p* = 0.001), and the general assessing of the physical fitness (*p* = 0.002). Men present a higher mean in all tests.

As for age, there is a correlation with the plate tapping test. ANOVA indicates that the age range from 15 to 17 years is statistically different (*p* = 0.001), in which 15-year-old teenagers have a higher mean (Table 4).

## 7. ALPHA-FITNESS Battery

The estimate for Spearman's rank correlation coefficient (Table 5) was carried out for the gender and age physical fitness variables for the modified and extended ALPHA-FITNESS battery, where it can be seen that gender correlates with the standing broad jump (*p* = 0.000), the handgrip test (*p* = 0.006), skinfold thickness (*p* = 0.000), and waist circumference (*p* = 0.003), while age was not statistically significant in any of the variables.

With the ANOVA single factor tests it was concluded that the variance of homogeneity was not met for the handgrip variable, so the Kruskal Wallis test was used instead of the ANOVA test; according to the statistical analysis, gender was related to the measure of variables, in which men have a higher mean in the standing broad jump (*p* = 0.004). The same can be observed in the handgrip test (*p* = 0.006). However, this is the opposite for the skinfold thickness (*p* = 0.000) and waist circumference (*p* = 0.005) (Table 6).

According to the Spearman correlation test, the BMI is directly related to the subscapular skinfold (*p* = 0.000), triceps skinfold (*p* = 0.000), and waist circumference (*p* = 0.000) (Table 7).

As for the analysis of the multiple comparison post hoc test (Dunnett's) as well as the corresponding homogeneity tests, it can be seen that the mean of the percentage of fat in such skinfolds and waist circumference is higher as the IMC is also higher (Table 8).

## 8. Comparison of Homologous Variables of the EUROFIT and the Extended, Modified ALPHA-FITNESS Batteries

The correlation of variables of the EUROFIT and the extended, modified ALPHA-FITNESS: The homologous variables are the standing broad jump, handgrip, and Course-Navette; when the comparison of this variables was carried out using Student's *t*-test, it can be observed that there are significant differences for the standing broad jump (*p* = 0.000) and the Course-Navette test (*p* = 0.000) (Table 9).

There is a significant difference in the means of the standing broad jump and Course-Navette variables, so it can be argued that the results of the variables at this stage, for Mexican teenagers from 15 to 17 years old, are statistically different for the EUROFIT and the extended, modified ALPHA-FITNESS batteries.

## 9. Discussion

The objective of this study was to determine the influence of weight, age, and gender on physical fitness assessed with the components of trustworthy, sensitive, and economical batteries whose validity, reliability, applicability, and relation to health have been proven in children and teenagers [13]. According to the initial hypotheses, overweight and obesity might have reduced the physical fitness levels of subjects. However, the same can be observed in those with normal weight and even those with low weight. Although it is well known that children or teenagers with overweight or obesity are predisposed to be adults with overweight or obesity, the “fat but fit” paradigm is also well known; independently of a high body fat percentage, subjects may have good physical fitness. There could also be the “fit but unhealthy” paradigm of which there are not enough reports and it is exactly what we encountered in this study, where more than 50% of the subjects are in the normal weight category and did not outstand in the healthy physical fitness tests.

In this study, a higher percentage belongs in the normal weight category; there is research that reports a higher number of subjects with normal weight; regarding the rest who report a higher amount of hours of physical activity and a better BMI [14], although it does not report if they also present healthy physical fitness, it is well known that the BMI is often considered an indicator of body fat when it actually measures the excess of weight instead of the excess of fat [15]; unlike other studies [16], in this study the prevalence of overweight/obesity is higher in women than in men. Women had higher results on skinfold thickness and waist circumference. This data is similar to that of another study on teenagers from Spain [17] and another one in Colombia [18, 19], where there are less men who suffer from overweight/obesity according to BMI and less percentage of fat tissue.

The fact that there is an evident sexual dysmorphism, characterized by higher percentages of fat values among women on all the groups that were analysed, is partially due to sexual development, socioeconomic status, diet, physical activity levels, or neurohormonal or ethnic factors of the specific population [20]. It has been observed that body fat percentage by electric bioimpedance in Mexican-American teenagers shows higher values in fat mass than Caucasians and African Americans non-Hispanics of all ages [20, 21].

As for the tests assessment of both genders, male participants had, overall, a better performance in the physical fitness tests, especially in the handgrip, standing broad jump tests, flamingo balance, and torso flexion, where there are similarities with Spanish [17, 22] and Venezuelan [23] teenagers. This profile shows, once more, that males, who have a better overall performance, have more abilities in the physical-motor area. Similar results can be found in studies with larger samples in Colombia, Guatemala, Chile, and other countries [18, 24–26].

The handgrip test results indicate that the aerobic capacity of 97% of the participants is within or above the 50th percentile, which is equivalent to the data obtained in studies in Spain. In relation to Spanish and Australian studies, the aerobic capacity levels of this study were higher in male participants [27–30]. This characteristic was not higher with older participants, for it reached its peak with 15-year-old participants.

Unlike a recent study with Colombian teenagers and children where the relation between muscular performance and early cardiovascular risk and physical wellness was studied through the standing broad jump and handgrip tests [31], teenagers in our study have higher handgrip levels, regardless of their body build, age, and gender, different from standing broad jump, where they have higher results.

There is evidence from observational studies that a low level in muscular force is an independent risk factor for cardiometabolic disease, above other risk factors such as hypertension or overweight and obesity [31–33]. There is also the 4-year PURE (Prospective Urban Rural Epidemiology) [34] study which shows that a decrease of five kilograms out of the grip strength per handgrip has an inverse association with cardiovascular mortality and ischemic coronary events; this strength decrease is also associated with dyslipidaemia [35], arterial stiffness [36], obesity [37], and less cardiorespiratory capacity [38]. In this study, despite the fact that the participant's cardiorespiratory capacity was low, different from hand grip strength, it is suggested that the participants have a preventive, protective factor against these risks, for their hand grip strength was high, regardless of variables such as age, gender, and BMI. However, nowadays there are few comparative studies of the health in children and the teenagers with muscular competence [39, 40].

There are studies that prove that a better muscular performance is related to better physical fitness [31]. Likewise, there is another study which shows that a low aptitude muscular level is connected to more probabilities of gaining at least 10 kg, regardless of the BMI and the cardiorespiratory fitness in men and women [37]. What this study highlights is that participants with a good hand grip strength level have a protective factor against metabolic diseases and lower their probabilities of gaining weight. As for the results of our study and the referenced studies, the muscular component might be considered as a cardiovascular health indicator with high discriminatory power [41–43].

Regarding the results of this study, in which participants had high levels of hand grip strength, it seems that a strength training programme might be enough to achieve a healthy cardiometabolic profile in healthy [44] and overweight [45] subjects. Muscular strengthening might also be an alternative for those who, due to medical reasons, cannot take part in the specific [46] cardiorespiratory exercise, and also as a prevention of metabolic diseases, dementia, arterial hypertension, atherosclerosis, sarcopenia, and obesity [47, 48].

An innovative result of this study is that, of the homologue components of the studied batteries, where meaningful differences are found in the standing broad jump test and the Course-Navette tests, no similar reports can be found. It is believed that these differences are found because the results of the tests are classified differently in relation to the score obtained.

Similarly, in the speed plate tapping test in which 15-year-old teenagers have a higher mean, this is likely to be because they are the group with the highest percentage of normal weight, with a suitable BMI according to their sex and age, which might allow higher train speed over the other two groups. It is also possible that in this test sex could influence.

There is a recent study, where, as in this study, participants were classified into three groups according to BMI: normal weight, overweight, and obese; then, six phenotypes were defined based on BMI and metabolic health: metabolically healthy and metabolically unhealthy. Interestingly, from what is observed in this study during a 12-year follow-up, even subjects with normal weight, but metabolically unhealthy, all metabolically unhealthy phenotypes, showed increased risk of cardiovascular diseases (CVD) events. This finding did not change after further adjustments, among which is physical activity. That is to say, metabolically healthy subjects, regardless of BMI category, were not at increased risk for incident CVD [49].

One of the limitations of this study was that there was a shortage of homogenous subjects to compare in the groups and that this study is a cross-sectional study, so it cannot determine causality, only association. Variables such as socioeconomic status or objective physical activity levels, which have a big influence on physical fitness, were not included.

The strength of this study is that it was carried out using perfectly validated batteries for teenagers. It is also important to mention that this study was carried out taking into account both genders, most of them in the normal weight category, which offers new perspectives on healthy physical fitness of Mexican teenagers.

## 10. Conclusions

The results of this study do not show definite conclusions about the BMI, gender, and age on physical fitness due to the previously mentioned limitations. It rather sets the basis for further research. Generally speaking, once there are homogeneous BMI, gender, and age groups and bigger samples, the research could be added to clinical analysis, thus monitoring the dynamics of each variable.

The general physical fitness assessment of Mexican teenagers in an average category was the main focus of this study, which allows us to think that one of the main components of physical condition and the most directly related to health, such as cardiorespiratory capacity, is at a low-regular level, which might be influenced by low physical activity, a sedentary lifestyle, and inadequate eating habits. It is therefore important to do more research on lifestyle, eating habits, and physical activity of Mexican teenagers, because being the first place in obesity and overweight puts us in more danger of suffering from disease and death at an early age or even suffering from handicaps, which may mean more potentially wasted years. Moreover, high public health expenses for being admitted into a hospital, permanent medicine without apparent use, and the consequences or complications of an unhealthy physical fitness also affect the economic situation of the families and the productivity of society, lower the quality of life, and increases the risk of health problems.

Derived from this study, there are new hypotheses related to the paradigms of physical fitness, above all in Mexico: “fat but fit” and “fit but unhealthy,” to the metabolic memory of the Mexican population, and the genetics of the country, for in this study, due to the poor physical fitness reported by teenagers with normal weight; it may be considered that they have the same risk of suffering from metabolic chronic degenerative diseases as people who suffer from overweight or obesity. This might mean that a healthy weight is not enough to prevent metabolic diseases but that regular physical activity may be necessary, especially at an early age, for this would allow us to minimize the consequences or complications that are the result of an unhealthy physical fitness.

## Figures and Tables

**Table 1:** Body weight status distribution based on the BMI category according to age and gender in the sample (*n* = 150).

Age	Weight status	Gender				Total	
		Men		Women			
		*N*	Weight status%	*N*	Weight status%	*N*	Weight status%
15	Normal	15	71.4%	24	80%	39	76.4%
	Low weight	1	4.7%	1	3.3%	2	3.9%
	Overweight	5	23.8%	4	13.3%	9	17.6%
	Obesity	0	0%	1	3.3%	1	1.9%

16	Normal	20	76.9%	24	68.5%	44	72.1%
	Low weight	3	11.5%	3	8.5%	6	9.8%
	Overweight	1	3.8%	5	14.2%	6	9.8%
	Obesity	2	7.6%	3	8.5%	5	8.1%

17	Normal	14	87.5%	15	68.1%	29	76.3%
	Low weight	2	12.5%	2	9.0%	4	10.5%
	Overweight	0	0%	3	13.6%	3	7.8%
	Obesity	0	0%	2	9.0%	2	5.2%

76.4% the normal weight category were 15 years old. Group of 16-year-olds is the one where most of the women (14.2%) suffer from overweight and in group of 17-year-olds 9% suffer obesity. There are no overweight and obesity men in the group of those who are 17 years old.

**Table 2:** Spearman correlation between both genders and age versus the results of the EUROFIT battery assessed variables in 15-to-17-year-old Mexican teenagers (*n* = 150).

		Flam. Bal.^a^	Plate Tapp.^b^	Tor. Flex.^c^	Stan. B.J.^d^	Hand.^e^	30 Abs.^f^	B.A H..^g^	10 × 5^h^	C-N^i^	Height	Weight	BMI^j^	Ov.P. F.^k^
Gender	*r* ^*∗*^	0.226^*∗*^	0.156	0.184^*∗*^	0.295^*∗∗*^	0.084	0.122	0.083	−0.030	−0.043	0.641^*∗∗*^	0.330^*∗∗*^	0.070	0.242^*∗∗*^
	*p* value	**0.005**	0.056	**0.025**	**0.000**	0.308	0.138	0.313	0.717	0.599	**0.000**	**0.000**	0.397	**0.003**
Age	*r* ^*∗*^	0.118	−0.310^*∗∗*^	−0.042	−0.013	−0.115	0.126	−0.045	−0.071	−0.016	0.044	−0.038	−0.075	−0.034
	*p* value	0.150	**0.000**	0.607	0.875	0.163	0.124	0.587	0.386	0.849	0.595	0.640	0.362	0.680

The shown values are the values of *r*^*∗*^ Spearman's rank correlation coefficient; ^*∗*^level of significance 0.01 and ^*∗∗*^level of significance 0.05. *p* value: significance value (*p* ≤ 0.05). ^a^Flamingo balance. ^b^Plate tapping test. ^c^Torso flexion. ^d^Standing broad jump. ^e^Handgrip. ^f^30′ abs. ^g^Bent arm hang. ^h^10 × 5 m shuttle run. ^i^Course-Navette. ^j^Body mass index. ^k^Overall assessment of the physical fitness.

**Table 3:** Kruskal Wallis parametric test results and single factor ANOVA between both genders and the variables of the EUROFIT battery in 15-to17-year-old Mexican teenagers (*n* = 150).

Variables	Gender	Mean ± SD	Levene test (Sig)	Kruskal-Wallis	ANOVA between groups			*p* value
					Quadratic mean	*F*	Sig.	
FLA. BAL.^a^	M	4.13 ± 1.00	12.86 (0.000)	0.006	7.23	8.99	0.003	**0.006**
	H	4.57 ± 0.712						
TORSO FLEX^b^	M	3.45 ± 1.42	5.93 (0.016)	0.025	9.88	5.55	0.20	**0.025**
	H	3.97 ± 1.19						
HOR. JUMP^c^	M	1.15 ± 0.495	24.87 (0.000)	0.000	3.53	9.91	0.002	**0.000**
	H	1.46 ± 0.714						
HEIGHT. (cm)	M	156.70 ± 6.87	2.90 (0.090)		4391.12	107.09	0.000	**0.000**
	H	167.66 ± 5.68						
WEIGHT (Kg)	M	55.63 ± 11.84	0.181 (0.671)		1672.48	12.54	0.001	**0.001**
	H	62.40 ± 11.13						
P. F. A.^e^	M	2.15 ± 0.581	3.81 (0.053)		3.18	9.68	0.002	**0.002**
	H	2.44 ± 0.562						

The data is presented as the mean ± standard deviation (SD). *p* = significance value (*p* ≤ 0.05). ^a^Flamingo balance test. ^b^Torso flexion. ^c^Standing broad jump. ^e^Overall physical fitness assessment.

**Table 4:** ANOVA single factor test results for age and the plate tapping of the EUROFIT battery in Mexican teenagers from 15 to 17 years old (*n* = 150).

Variable	AGE	Mean ± SD	Levene test	Sig.	ANOVA between groups			*p* value
					Quadratic mean	*F*	Sig.	
Plate tapping test	15	3.67 ± 1.55	0.112	0.894	17.86	7.38	0.001	**0.001**
	16	3.06 ± 1.59						
	17	2.38 ± 1.47						

The data is presented as the mean ± standard deviation (SD).

**Table 5:** Estimate for Spearman's rank correlation coefficient of age and gender versus the results of the studied variables of the modified and extended ALPHA-FITNESS battery for Mexican teenagers from 15 to 17 years old (*n* = 150).

		Stan. B. J.^a^	Hand.^b^	4 × 10 m^c^	Course-N^d^	Tric. Sk.^e^	Sub. Sk.^f^	BMI^g^	Waist Circ.^h^
Gender	*r* ^*∗*^	0.284^*∗∗*^	0.193^*∗∗*^	0.081	0.131	−0.120	−0.322^*∗∗*^	−0.129	−0.219^*∗∗*^
	*p* value	**0.000**	**0.006**	0.327	0.111	0.112	**0.000**	0.087	**0.003**
Age	*r* ^*∗*^	0.020	0.058	−0.127	−0.095	−0.119	−0.063	−0.102	0.009
	*p* value	0.811	0.413	0.122	0.249	0.118	0.408	0.177	0.902

The shown values are *r*^*∗*^ values of the estimate for Spearman's rank correlation coefficient; ^*∗∗*^significance value 0.05. *p* = significance value (*p* ≤ 0.05). ^a^Standing broad jump. ^b^Handgrip. ^c^4 × 10 m shuttle run. ^d^Course-Navette (cardiorespiratory capacity). ^e^Triceps skinfold. ^f^Subscapular skinfold. ^g^Body mass index. ^h^Waist circumference.

**Table 6:** Kruskal-Wallis test results and ANOVA single factor between both genders and the variables of the modified, extended ALPHA-FITNESS test for Mexican teenagers from 15 to 17 years old (*n* = 150).

Variables	Gender	Mean ± SD	Levene Test (Sig)	Kruskal-Wallis	ANOVA between groups			*p* value
					Quadratic mean	*F*	Sig.	
Standing B. J.^a^	F	1.61 ± 0.992	0.033 (0.856)		8.63	8.50	0.004	**0.004**
	M	2.10 ± 1.02						
Handgrip	F	4.81 ± 0.766	25.53 (0.000)	0.006	1.91	5.87	0.016	**0.006**
	M	5.00 ± 0.00						
Sub. Sk. (mm)^b^	F	3.68 ± 1.06	0.955 (0.330)		27.43	20.43	0.000	**0.000**
	M	2.88 ± 1.27						
Waist Cir (cm)^c^	F	3.38 ± 1.20	3.33 (0.070)		11.04	8.06	0.005	**0.005**
	M	2.88 ± 1.12						

The data is presented as mean ± standard deviation (SD). *p* = significance value (*p* ≤ 0.05). ^a^Standing broad jump. ^b^Subscapular skinfold. ^c^Waist circumference.

**Table 7:** BMI correlation versus the results of the studied variables in the modified, extended ALPHA-FITNESS battery test for Mexican teenagers from 15 to 17 years old. (*n* = 150).

		Gender	Age	S.B.J.^a^	Hand.^b^	4 × 10 m.^c^	Cou-Na.^d^	Triceps skinfold	Subscapular skinfold	Waist Circum^e^.
	*r* ^*∗*^	−0.129	−0.102	−0.141	0.075	−0.084	−0.156	0.653^*∗∗*^	0.716^*∗∗*^	0.621^*∗∗*^
BMI	Valor de *p*	0.087	0.177	0.086	0.323	0.304	0.057	**0.000**	**0.000**	**0.000**

The values shown are variables of estimate for Spearman's rank correlation coefficient *r*^*∗*^. ^*∗∗*^Significance value 0.05. *p* = significance value (*p* ≤ 0.05). ^a^Standing broad jump. ^b^Handgrip test. ^c^4 × 10 m shuttle run. ^d^Course-Navette (cardiorespiratory test). ^e^Waist circumference.

**Table 8:** Kruskal-Wallis test results and single factor ANOVA test between the BMI and anthropometry variables of the extended, modified ALPHA-FITNESS battery test for Mexican teenagers from 15 to 17 years old (*n* = 150).

Variables	BMI^a^	Mean ± SD	Levene Test (Sig)	Kruskal-Wallis	ANOVA between gruops			*p* value
					Quadratic mean	*F*	Sig.	
TRIC. SKIN.^b^	VL^e^	1.60 ± 0.966	1.04 (0.388)		24.82	34.15	0.000	**0.000**
	L^f^	2.14 ± 0.891						
	A^g^	2.67 ± 0.920						
	H^h^	3.34 ± 0.653						
	VH^i^	4.27 ± 0.785						

SUB. SKIN.^c^	VL	1.10 ± 0.316	7.34 (0.000)	0.000	36.88	55.88	0.000	**0.000**
	L	2.54 ± 0.999						
	A	3.13 ± 0.859						
	H	3.88 ± 0.907						
	VH	4.83 ± 0.379						

WAIST CIRCUM.^d^	VL	1.80 ± 1.31	0.953 (0.435)		24.35	27.37	0.000	**0.000**
	L	2.39 ± 0.875						
	A	2.93 ± 0.905						
	H	3.63 ± 0.793						
	VH	4.45 ± 1.09						

The values are presented as the mean ± standard deviation (SD). *p* = significance value (*p* ≤ 0.05). ^a^Body mass index. ^b^Triceps skinfold. ^c^Subscapular skinfold. ^d^Waist circumference. ^e^Very low. ^f^Low. ^g^Average. ^h^High. ^i^Very high.

**Table 9:** Comparison of the homologous variables of the EUROFIT and the extended, improved ALPHA-FITNESS batteries for Mexican teenagers from 15 to 17 years old (*n* = 150).

Variables	Mean ± SD	Paired sample correlations (Sig)	Paired samples test (Student's *t*-test)		*p* value
			MEAN ± SD	*t*	
Standing broad jump	1.28 ± 0.614	0.654 (0.000)	−0.533 ± 0.783	−8.34	**0.000**
Standing broad jump	1.81 ± 1.03				
Handgrip	4.90 ± 0.588	0.621 (0.000)	0.027 ± 0.530	0.616	0.539
Handgrip	4.87 ± 0.627				
Course-Navette	1.31 ± 0.612	0.711 (0.000)	−0.813 ± 0.806	−12.36	**0.000**
Course-Navette	2.12 ± 1.11				

The values are presented as the mean ± standard deviation (SD). *p* = significance value (*p* ≤ 0.05).
